# Unconditional cash transfers and maternal substance use: findings from a randomized control trial of low-income mothers with infants in the U.S.

**DOI:** 10.1186/s12889-022-12989-1

**Published:** 2022-05-05

**Authors:** Paul Y. Yoo, Greg J. Duncan, Katherine Magnuson, Nathan A. Fox, Hirokazu Yoshikawa, Sarah Halpern-Meekin, Kimberly G. Noble

**Affiliations:** 1 School of Education, University of California, Irvine, 401 E. Peltason Drive, Suite 3200, Irvine, CA 92697 USA; 2 Sandra Rosenbaum School of Social Work, University of Wisconsin-Madison, 1350 University Ave, Madison, WI 53706 USA; 3 College of Education, University of Maryland, College Park, 3119 Benjamin Building, College Park, MD 20742 USA; 4 Steinhardt School of Culture, Education, and Human Development, New York University, 82 Washington Square E, New York, NY 10003 USA; 5 School of Human Ecology & La Follette School of Public Affairs, University of Wisconsin-Madison, 1300 Linden Dr., Madison, WI 53706 USA; 6 Teachers College, Columbia University, 525 West 120th Street, New York, NY 10027 USA

**Keywords:** Poverty, Cash transfer, Child Allowance, Substance use, Randomized Control Trial

## Abstract

**Background:**

Policy debates over anti-poverty programs are often marked by pernicious stereotypes suggesting that direct cash transfers to people residing in poverty encourage health-risking behaviors such as smoking, drinking, and other substance use. Causal evidence on this issue is limited in the U.S. Given the prominent role of child allowances and other forms of cash assistance in the 2021 American Rescue Plan and proposed Build Back Better legislation, evidence on the extent to which a monthly unconditional cash gift changes substance use patterns among low-income mothers with infants warrants attention, particularly in the context of economic supports that can help improve early environments of children.

**Method:**

We employ a multi-site, parallel-group, randomized control trial in which 1,000 low-income mothers in the U.S. with newborns were recruited from hospitals shortly after the infant’s birth and randomly assigned to receive either a substantial ($333) or a nominal ($20) monthly cash gift during the early years of the infant’s life. We estimate the effect of the unconditional cash transfer on self-report measures of maternal substance use (i.e., alcohol, cigarette, or opioid use) and household expenditures on alcohol and cigarettes after one year of cash gifts.

**Results:**

The cash gift difference of $313 per month had small and statistically nonsignificant impacts on group differences in maternal reports of substance use and household expenditures on alcohol or cigarettes. Effect sizes ranged between − 0.067 standard deviations and + 0.072 standard deviations. The estimated share of the $313 group difference spent on alcohol and tobacco was less than 1%.

**Conclusions:**

Our randomized control trial of monthly cash gifts to mothers with newborn infants finds that a cash gift difference of $313 per month did not significantly change maternal use of alcohol, cigarettes, or opioids or household expenditures on alcohol or cigarettes. Although the structure of our cash gifts differs somewhat from that of a government-provided child allowance, our null effect findings suggest that unconditional cash transfers aimed at families living in poverty are unlikely to induce large changes in substance use and expenditures by recipients.

**Trial Registration:**

Registered on Clinical Trials.gov NCT03593356 in July of 2018.

**Supplementary Information:**

The online version contains supplementary material available at 10.1186/s12889-022-12989-1.

## Background

Poverty is driven by underinvestment in communities, systematic discrimination, and other structural disadvantages. And though most Americans express a desire to help people living in poverty, [1] derogatory stereotypes about poor adults, especially women with children, have led some to undermine support for government assistance. Katz [2] documents how the so-called “undeserving poor”— individuals living in poverty, particularly Black people with low income – have long been blamed for their plight and considered unworthy of government assistance.

These stereotypes of individuals living in poverty can hamper adoption of anti-poverty policies and programs. Gilens’s [1] analysis of public opinion in the US finds that negative perceptions of people who receive governmental assistance in general, and of Black people in particular, are significant barriers to providing more government assistance and services. Legislative action based on suspicion of drug and other substance use on the part of welfare recipients has been commonplace since the 1996 welfare reform, which allowed states to test people for illicit drugs and impose sanctions if they received governmental support (see P.L. 104–193 Sect. 902.) [3]. By 2017, at least 15 states had passed legislation requiring drug testing of welfare recipients [4].

Using data from a cash transfer policy experiment, we explore one piece of this policy debate with implications for children’s healthy development [5–7] – whether a monthly unconditional cash transfer affects substance use and related spending among low-income mothers of infants.

Although public concerns over substance use often focus on individuals living in poverty, usage rates among both economically advantaged and disadvantaged Americans vary widely depending on the type of substance and population being considered. For example, alcohol use is much more prevalent among affluent than low-income adults, while cigarette use is more common among lower-income adults. In the U.S. between 2011 and 2014, 73.9% of women with family incomes at least four times the poverty line reported drinking alcohol regularly, compared with 43.1% of women living in households with incomes below the poverty line [8]. In the case of cigarette smoking, 23.9% of women living in poverty reported regular cigarette use compared with 9.5% of more affluent women. [9] Researchers have also found a very low prevalence of substance use disorders among individuals receiving governmental assistance [10].

Policy debates include the issue of whether increased government assistance leads to considerable increase in expenditures on alcohol, cigarettes, and related substances. Although economic studies generally show that household expenditures on most goods and services increase as income increases [11], it is not clear whether this applies to alcohol, cigarettes, and related substance expenditures among low-income mothers. One recent meta-analysis using national aggregate data as well as data from household surveys found that a 10% increase in income was generally associated with between a 5% and 10% increase in spending (i.e., an “income elasticity” of 0.5-1.0) on alcohol [12]. Estimates of the corresponding income elasticity of adult expenditures on cigarettes are more variable, but most suggest a smaller increase in spending on cigarettes relative to alcohol expenditures [13]. With regard to the third drug measured in our survey – opioids – we know of no studies estimating the sensitivity of opioid use or expenditures to income changes.

Quasi-experimental studies of expenditure changes [14, 15] and health behaviors [16–21] in response to the 1990s welfare reforms and expansions of the Earned Income Tax Credit (EITC) show no significant impacts of increased benefits on alcohol and tobacco expenditures and either no significant impacts on alcohol and tobacco use or significant reductions in maternal smoking for low-income women. Quasi-experimental studies of expenditure changes in the UK Family Expenditure Survey [22, 23] in response to the introduction of a Child Benefit cash assistance program show a mix of positive and negative effects on household alcohol expenditures for low-income families.

The most rigorous evidence on substance use changes in response to cash transfers comes from randomized trials [24, 25] conducted in low- and middle-income countries. Many of these experiments found that cash transfer programs reduced alcohol and tobacco consumption, while some found null effects. One possible explanation for reductions is that cash transfers may reduce economic strain, which can lead to subsequent stress-induced substance use and abuse.

To address these issues in the U.S. context, we draw data from an ongoing clinical trial in which low-income mothers with newborns were randomly assigned to receive either a substantial or much smaller monthly unconditional cash gift. We use these data to estimate the impacts of a monthly unconditional cash transfer on maternal substance use and household expenditures on alcohol and cigarettes across the first year of the infants’ lives. This study is particularly timely, in light of the fact that, as detailed below, the unconditional cash gifts in our clinical trial share some but not all features of the Child Tax Credit provisions of the American Rescue Plan and the Build Back Better legislation passed by the U.S. House of Representatives in November, 2021.

## Methods

### Overview of baby’s first years randomized control trial

Our data come from the Baby’s First Years (BFY) study, an ongoing multi-site parallel-group randomized control trial in which monthly unconditional cash gifts were disbursed to low-income mothers with newborn infants. The overall goal of the BFY study is to understand how enhanced economic resources shape children’s cognitive and socioemotional development, family life, and child and maternal health [26].

Between May, 2018 and June, 2019, the BFY interviewers recruited 1,051 mothers^1^ with incomes below the federal poverty threshold who gave birth at 12 hospitals in four metropolitan areas (New York City, the greater New Orleans metropolitan area, the greater Omaha metropolitan area, Minnesota’s Twin Cities). To participate, mothers had to be at least 18 years old, speak either English or Spanish, have newborns not requiring intensive care, and have newborns who would be discharged into their custody. Baseline data were collected in the hospitals shortly after the infant’s birth. The mothers were then offered the opportunity to receive a monthly unconditional cash gift. The 1,003 mothers who agreed to receive the cash gift were randomly assigned (see Supplemental Materials for details) into two groups within each site to receive different monthly cash gifts disbursed through a debit card for the first 52 months of their child’s life.

The debit card was activated at the hospital and the cash gift has been automatically disbursed each month on the day of the child’s birthdate. The debit card is branded with a “4MyBaby” logo, and each month mothers receive a text message reminder that the cash is available for use. Mothers in the high-cash gift group (*n* = 400) receive $333 per month for 52 months, while mothers in the low-cash gift group (*n* = 600) receive a monthly payment of $20 per month. Interviewers could not be blinded to treatment status at the point of hospital recruitment, although they were not informed (for new interviewers) or reminded (for continuing interviewers) of mothers’ treatment statuses at the time of the age-1 interview. Recruited mothers were informed of the two different monthly cash gift amounts at the conclusion of the hospital visit.

Of the 1,003 mothers who were randomized, three notified their interviewer within two days after completing the baseline interview that they wanted to withdraw from the study and stop receiving the cash gifts. BFY’s final sample comprises 1,000 mother-infant dyads, with 400 allocated to the high-cash gift group and 600 allocated to the low-cash-gift group. More details about the BFY study design, including the baseline CONSORT diagram, can be found in Noble et al. [27], and the preregistration details can be found on clinicaltrials.gov under identifier NCT03593356 [28].

**Fig. 1:**
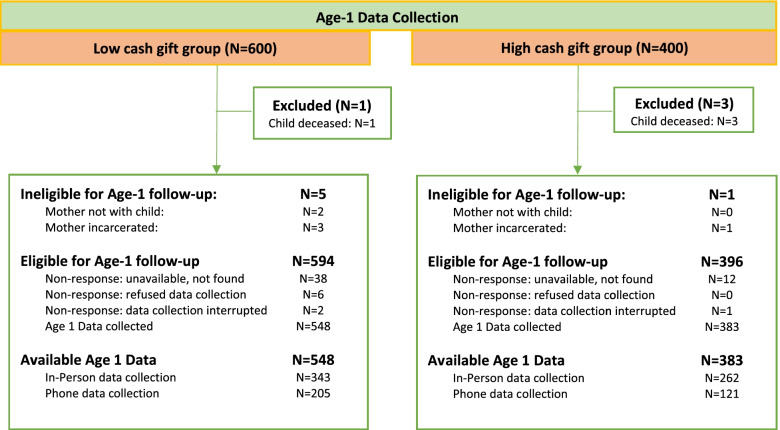
Age-1 CONSORT diagram

Between July, 2019, and July, 2020, BFY interviewers contacted as many of the 1,000 study participants as possible around the time of their children’s first birthdays. By the end of the field period, the BFY team had completed interviews with 931 of the 1,000 mothers. Adjusting the denominator for infant deaths (4), mother-child separations (2), and maternal incarcerations (4), the age-1 response rate was 94.0% (see Fig. 1 for the Age-1 CONSORT diagram and Noble et al. (2021) for the baseline CONSORT diagram). Because of the COVID-19 pandemic, BFY paused all in-home data collection on March 13, 2020 and, two days later, began administering telephone interviews. By the end of the age-1 fielding period, 597 of the age-1 interviews with valid substance use data had been conducted in person, and 930 had at least one substance use or expenditure outcome measured in person or over the phone. A complete accounting of field procedures is provided with materials in our baseline public data deposit [26] and on the study’s website [29].

### Measures of substance use and expenditures

During baseline interviews (which occurred shortly before random assignment), mothers reported how often they smoked cigarettes and drank alcohol, as well as the amount of alcohol or cigarettes consumed per occasion during the 3 months prior to pregnancy and during each trimester. A limitation of these data is that mothers were not asked questions about substance use disorders in either the baseline or age-1 interviews.

When their child was approximately one year old, mothers reported on their frequency of smoking, drinking, and opioid use, and the amount of alcohol consumed per drinking occasion in the past year. We preregistered two age-1 substance use outcomes: an additive 2-item index of the frequency of alcohol use and cigarette use, each on a 5-point response scale (0: never in last year; 1: less than 1 time per month; 2: several times per month; 3: several times per week; 4: every day) with a total range from 0 to 8; and frequency of opioid use on the same 5-point scale. In our main analysis, we estimate effects on this index outcome and its individual components.

As the preregistered outcomes are on an ordinal scale without equal intervals, we explored the robustness of our results by converting the ordinal scale into continuous measures with equal intervals. Specifically, we created an estimate of the number of substance use occasions per week (i.e., number of smoking, drinking, or opioid use occasions per week) by taking the mid-point of the ordinal frequency categories. For example, we assumed that mothers who reported drinking several times per week had 3.5 drinking occasions per week, which is the mid-point of drinking 1 time per week and 6 times per week. Estimates were not sensitive to lower or higher bound assumptions on conversion (e.g., converting several times per week into 2 or 5 times per week). To create an estimated number of alcoholic drinks per week, we took the product of the estimated number of drinking occasions per week and the typical number of drinks per occasion reported. We analyzed the effects on these continuous measures as a robustness check of the main estimates, and present them in Table S.A3 of Additional File 1.

Mothers also reported how many packs of cigarettes were purchased and how many dollars were spent on alcoholic beverages for the entire household in an average week during the most recent month prior to the age-1 visit. We approximated the dollar expenditures on cigarettes by taking the product of the number of cigarette packs purchased and the average cost of a cigarette pack in the respective metropolitan area, as reported by the Centers for Disease Control and Prevention [30]. The average cost of a pack of cigarette in 2019 for the four states in BFY were: LA -$6.08, MN -$9.13, NE-$5.78, NY-$10.53. Alcohol and cigarette expenditure outcomes were each winsorized at the 99th percentile to adjust for extreme values. We added the winsorized cigarette expenditures and alcohol expenditures to create a summary substance expenditure measure. We estimated effects on the summary measure and its components in our main analysis.

The more sensitive questions in the survey, including those about maternal tobacco, alcohol, and opioid use, were asked using pre-recorded audio played over headphones (audio computer assisted self-interviewing) rather than directly by the interviewer. As this procedure was not possible over the phone, these substance use measures are available only for the portion of the sample interviewed in person prior to the onset of the pandemic. This procedural change limits the analysis of treatment effects on maternal substance use measures to the subsample interviewed before the onset of the pandemic. We address the problem of missing data on these measures with various tests and weighting schemes (see Section B of Additional file 1 for details).

### Statistical analysis

We used the random assignment design of the BFY clinical trial to estimate the causal effect of additional $313-per-month cash gift payments for approximately 12 months (depending on the exact timing of the age-1 interview) on each of the substance use and substance expenditure measures. The effect was estimated by regressing each outcome measure on the treatment indicator and the four site indicators, since randomization occurred within site. Following our preregistration plan, we adjusted our estimates for the following baseline demographic child and family characteristics: mother’s age, mother’s years of completed schooling, household income, net worth, general health, depressive symptoms, race and ethnicity, marital status, number of adults in the household, number of other children born to the mother, number of cigarettes smoked per week during pregnancy, number of alcoholic drinks consumed during pregnancy, biological father living with the mother; child’s gender assigned at birth, birth weight, gestational age at birth. We adjusted also for age of child in months during data collection and whether the interview was conducted over the phone or in person (see Additional File 1 for details). Baseline controls for prior substance use adjust for departures from random assignment while also increasing the precision of the treatment effect estimate. We adjusted the standard errors using robust variance estimation techniques.

As per our preregistration, we addressed the possibility of false positives by estimating the statistical significance of the entire family (“familywise error rate”) of outcomes using step-down resampling methods developed by Westfall and Young [31]. For the Westfall-Young adjustment, the substance use measures were placed into one family and the expenditure measures were placed into one family. We ran our analyses separately on two analytic samples: the full study sample with age 1 substance use data (*N* = 930) and the subsample of participants interviewed in their homes before the onset of the COVID-19 pandemic (*N* = 597).

Our sample of 930 participants provided 80% power to detect a minimum effect size of 0.186 standard deviations at the 0.05 alpha level on a two-tailed test. Our pre-pandemic sample of 597 participants provided 80% power to detect a minimum effect size of 0.225 standard deviations at the 0.05 alpha level on a two-tailed test. For substance use measures, we can detect as little as one additional drinking occasion per 11.5 weeks (0.1 more drinking occasions per week); one additional drink per 6.2 weeks (0.2 more drinks per week); one additional smoking occasion per 2.5 weeks (0.4 more smoking occasions per week); and one additional opioid use occasion per 12.3 weeks (0.1 more opioid use occasions per week). Regarding expenditures, we can detect as little as an $18.11 increase in alcohol and cigarette expenditures per month, which is 6% of the $313 monthly income difference between the high-cash and low-cash gift groups.

## Results

### Baseline characteristics of the sample

**Fig. 2:**
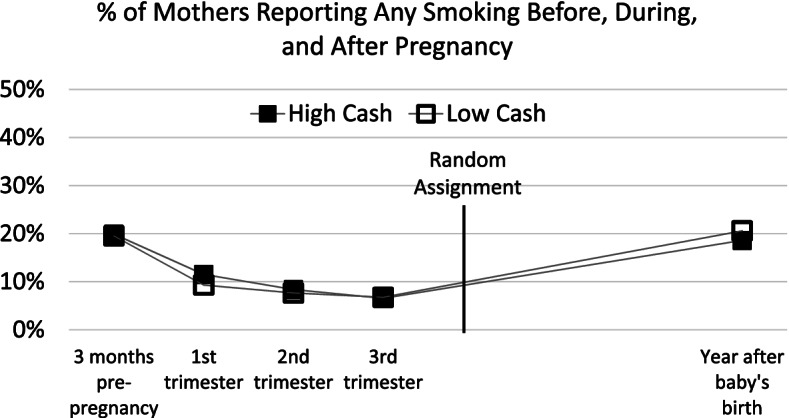
Percent of mothers reporting any smoking before, during, and after pregnancy

**Fig. 3:**
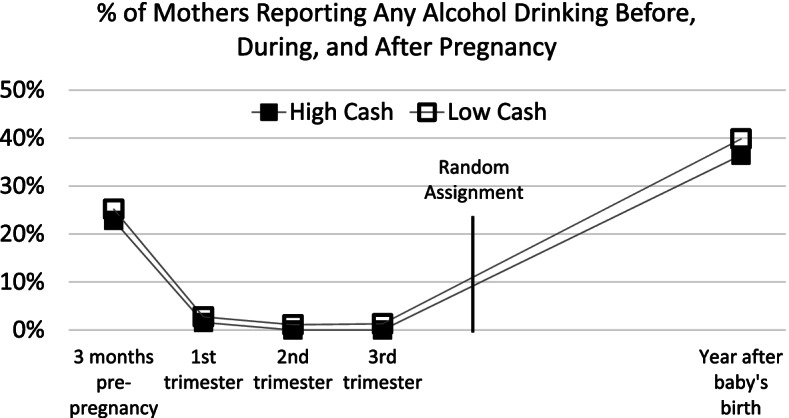
Percent of mothers reporting any alcohol drinking before, during, and after pregnancy

Baseline differences in demographic characteristics are shown in Table 1. To explore baseline equivalence on histories of substance use, we plot in Figs. 2 and 3 the percentage of mothers who reported during the baseline interview that they had ever smoked cigarettes or drunk alcohol in the 3 months prior to pregnancy and during each trimester of pregnancy. We also plot post-random assignment substance use estimates taken from the age-1 interview.

**Table 1:** Baseline characteristics of the full age-1 analytic sample (*N* = 930)

	Low-Cash Gift		High-Cash Gift		Std Mean Difference	
	Mean (sd)	N	Mean (sd)	N	Hedges’ g	Cox’s Index
**CHILD**						
Female	0.505	548	0.479	382		-0.063
Weight at birth(lbs)	7.136 (1.080)	547	7.107 (1.019)	381	-0.027	
Gestational age(weeks)	39.091 (1.234)	544	39.033 (1.253)	382	-0.047	
**MOTHER**						
Age at birth (years)	26.936 (5.838)	548	27.406 (5.761)	382	0.081	
Education(years)	11.86 (2.832)	541	11.921 (2.971)	380	0.021	
Race/Ethnicity						
White, non-Hispanic	0.106	548	0.081	382		-0.180
Black, non-Hispanic	0.387	548	0.442	382		0.137
multiple, non-Hispanic	0.042	548	0.031	382		-0.191
Other or unknown	0.044	548	0.024	382		-0.380
Hispanic	0.422	548	0.421	382		-0.002
Marital status						
Never married	0.418	548	0.497	382		0.193
single, living with partner	0.270	548	0.215	382		-0.182
married	0.215	548	0.212	382		-0.011
divorced/separated	0.046	548	0.029	382		-0.290
Other or unknown	0.051	548	0.047	382		-0.052
Health is good or better	0.880	548	0.924	382		0.306
Depression (CESD)	0.678 (0.443)	548	0.675 (0.448)	382	-0.007	
Cigarettes per week during pregnancy	4.676 (20.316)	544	3.118 (11.114)	379	-0.091	
Alcohol drinks per week during pregnancy	0.152 (1.662)	546	0.026 (0.390)	381	-0.096	
Number of children born to mother	2.420 (1.372)	548	2.529 (1.417)	382	0.078	
Number of adults in household	2.084 (0.984)	548	2.024 (0.968)	382	-0.062	
Biological father lives in household	0.411	548	0.351	382		-0.154
Household combined income	22313.093 (21282.425)	514	20979.771 (16030.742)	355	-0.069	
Household income unknown	0.062	548	0.071	382		0.088
Household net worth	-2187.560 (29365.991)	489	-3267.965 (20722.262)	342	-0.041	
Household net worth unknown	0.108	548	0.105	382		-0.019

The proportions of mothers who report drinking and smoking are similar across time in the high- and low-cash gift groups (see Table S.A1 of Additional File 1 for regression-adjusted estimates of these differences). Virtually none of the mothers in either group reported alcohol consumption during pregnancy, and less than 10% of both groups smoked during their second and third trimesters. As also seen in Figs. 2 and 3, the high-cash gift group includes a slightly smaller proportion of drinkers, but this difference is fairly stable over time. There are no statistically significant differences in the proportion of smokers across all time periods or in the continuous measures of frequency of substance use prior to or during pregnancy (i.e., estimated number of weekly cigarettes or alcoholic drinks). This suggests similar levels of substance use in the high- and low-cash gift groups during pregnancy, shortly after birth, and through the age 1 follow-up after mothers had received the cash gift for about 12 months. Although mothers were not asked about opioid use before and during pregnancy, their responses about opioid use at the time of the age 1 follow-up showed that similar and very small fractions engaged in such use (3.1% of mothers in the high-cash group and 3.5% of mothers in the low-cash group).

### Intent-to-treat analyses

To answer our question of whether the cash gift considerably increases the use of and household expenditures on substances, we turn to regression analyses. We present main causal effect estimates on substance use and expenditures at age 1 in Table 2, with full regression details provided in Table S.A2 of Additional File 1.

**Table 2:** Treatment effects on substance use and expenditures

	Maternal Substance Use Behavior				Family Substance Expenditure/Purchase per Week			
	Alcohol and Cigarette Use Index	Alcohol Use	Cigarette Use	Opioid Use	Alcohol and Cigarette (dollars)	Alcohol (dollars)	Cigarettes (dollars)	Cigarettes (N packs)
***A. Pre-pandemic Sample***								
Low-cash gift group mean (standard deviation)	1.112 (1.597)	0.475 (0.649)	0.635 (1.331)	0.076 (0.467)	12.435 (26.401)	3.412 (9.267)	8.976 (22.958)	1.288 (3.328)
Cash-gift treatment effect (standard error)	0.057 (0.118)	0.031 (0.055)	0.028 (0.096)	-0.031 (0.025)	0.050 (2.105)	1.174 (0.914)	-1.123 (1.737)	-0.144 (0.251)
Effect size	0.036	0.047	0.021	-0.067	0.002	0.127	-0.049	-0.043
* p*-value, unadjusted	0.628	0.580	0.769	0.211	0.981	0.199	0.518	0.566
* p*-value, adjusted	0.826	0.826	0.826	0.552	0.980	0.395	0.69	0.713
N	597	597	598	597	593	595	595	595
***B. Full Sample***								
Low-cash gift group mean (standard deviation)					9.997 (22.542)	3.432 (9.297)	6.582 (19.224)	0.925 (2.632)
Cash-gift treatment effect (standard error)					0.394 (1.493)	0.668 (0.705)	-0.296 (1.204)	-0.039 (0.160)
Effect size					0.017	0.072	-0.015	-0.015
* p*-value, unadjusted					0.792	0.344	0.806	0.809
* p*-value, adjusted					0.957	0.631	0.957	0.957
N					920	922	927	927
Preregistered hypothesis	YES	NO	NO	NO	NO	NO	NO	NO

Notes: Alcohol use, cigarette use, and opioid use are maternal self-report measures scored on a 0–4 point frequency ordinal scale (0: never in last year; 1: less than 1 time per month; 2: several times per month; 3: several times per week; 4: everyday). The Alcohol and Cigarette Use Index is a preregistered additive index of alcohol use and cigarette use, which ranges from 0–8. Adjusted *p*-values are Westfall and Young adjustments for multiple hypothesis testing. For the adjustment, substance use measures are placed into one family and expenditure measures are placed into one family. Effect size is the treatment effect divided by the standard deviation of low-cash gift group. + *p* < 0.10; * *p* < 0.05

We find no statistically significant differences between the high-cash gift group and the low-cash gift group in mothers’ use of alcohol, cigarettes, and opioids. The point estimates are very small, amounting to 0.06 more units on the combined alcohol and cigarette use index and 0.03 fewer units on the opioid use index on the preregistered ordinal frequency scale. Using more interpretable units (presented in Table A.3 of Additional File 1), the high-cash gift group is estimated to have 0.04 more smoking occasions per week on a base of 0.88 weekly smoking occasions; 0.02 more drinking occasions per week on a base of 0.10 weekly drinking occasions; 0.04 drinks per week on a base of 0.20 weekly drinks; and 0.06 fewer opioid use occasions per week on a base of 0.09 opioid use occasions, none of which are statistically significant at the 0.05 alpha level. The small negative effect on opioid substance occasions per week in Table S.A3 is marginally significant (β=-0.055, SE = 0.029), but it is not robust to Westfall-Young multiple-testing adjustments. In addition, we ran an ordered logit regression on the alcohol, cigarette, and opioid use measures on the 5-point frequency scale and find similarly null results (see Table S.A4). Taken together, these results indicate that we cannot reject the null hypothesis that the $313 difference in monthly unconditional cash transfers has no effect on the substance use of low-income mothers with infants.

While our estimation of the effect of the high-cash gift on substance use is limited to the sample of mothers interviewed before the onset of the pandemic, we are able to estimate the effect of the cash gift difference on household expenditures on alcohol and cigarettes for the full analytic sample. As shown in the bottom panel of Table 2, we find no statistically significant group differences in mothers’ reports of household expenditures on or purchase of cigarettes or alcohol. Table 2 also shows that treatment effects are similarly null when the results for the sample interviewed before the onset of the pandemic are compared with results for the full sample. We tested whether treatment effects on expenditure outcomes interacted with the subsample indicator and did not find a significant interaction.

Looking across all of the outcomes, effect sizes are + 0.047 or smaller for alcohol use, tobacco use, and alcohol expenditures, with none approaching conventional levels of statistical significance. In the case of opioid use and cigarette expenditures, the effect size estimates are negative (-0.067 and − 0.015, respectively), and also not statistically significant.

When converted to monthly amounts, the full-sample results in the bottom panel of Table 2 provide our best estimates of how much of the $313 difference in monthly cash gift payments made to the high-cash and low-cash gift groups is allocated to substance expenditures. Monthly cigarette expenditures are estimated to fall by $1.28 from a base of $28.52 (-4.5%), while monthly alcohol expenditures are estimated to increase by $2.89 from a base of $14.87 (+ 19.4%), with the combined alcohol and cigarette expenditures estimated to increase by $1.71 from a base of $43.32 (+ 3.9%). None of these estimates is close to conventional levels of statistical significance.

Another way of thinking about the size of these estimated effects is in terms of the health-policy-relevant hypotheses that substantial portions of the cash gifts will be spent on substances. However, the estimated $1.71 increase in monthly expenditures on alcohol and tobacco expenditures implies that less than 1% of the $313 monthly income difference between the high-cash and low-cash gift groups is spent on alcohol and tobacco.

In Tables S.A5 and S.A6 of Additional File 1, we ran impact analyses for subgroups that differ in reported substance use prior to randomization, including whether mothers reported any smoking and/or alcohol use during pregnancy or in the 3 months prior to becoming pregnant. We do not find any treatment effects on substance use or expenditures that vary significantly by prior substance use. We find a marginally significant positive treatment effect on family-level expenditures per week on alcohol (β = 1.544, SE = 0.802) for the subgroup with no prior alcohol use before or during pregnancy, suggesting that, among women who report no pre-pregnancy alcohol use, the high-cash gift group tended to have higher alcohol expenditures than the low-cash gift group. However, this effect was not robust to multiple comparison adjustment (Westfall-Young adj. *p*-value = 0.103). The interaction of treatment with prior alcohol use was statistically significant but not robust to multiple comparison adjustment (*p*-value = 0.029; Westfall-Young adj. *p*-value = 0.086).

## Discussion

The present study tested the impact of a monthly unconditional cash transfer to mothers of infants residing in low-income households. We find no evidence that an additional $313 per month in unconditional income has a substantial effect on maternal substance use or on household-level expenditures on alcohol or cigarettes. The findings are robust to adjustments for potential non-response bias and baseline differences in the analytic samples. They are also robust to whether or not mothers were interviewed prior to the onset of the COVID-19 pandemic. The finding that monthly unconditional cash gifts did not substantially increase expenditures on alcohol and tobacco is largely in line with evidence from cash transfer studies implemented in low- to middle-income countries and quasi-experimental studies of the Earned Income Tax Credit in the U.S.

Null results are not evidence of no effects, so how statistically informative is our null finding? We report all statistical power estimates in the above statistical analysis section, and we expand our discussion here for our finding regarding expenditures on alcohol and cigarettes. The study is powered to detect an increase of $18.11, which is large relative to typical monthly expenditures for the BFY sample – 42% of the low-cash gift group’s mean expenditures on alcohol and tobacco ($43.32) and 13% of the low-cash group mean with non-zero spending on substances ($138.41). Since the monthly cash gift difference amounts to a 17% increase in household income, the 42% minimum detectable proportionate increase in total expenditures translates into an income elasticity of 2.4, which is considerably larger than the 0.5 to 1.0 income elasticity estimates found in the economics literature [11–13]. While the study is not powered to detect small increases in substance expenditures, it is well powered to test and reject health-policy-relevant hypotheses that substantial portions of the cash gifts will be spent on substances. Specifically, the $18.11 minimum detectable effect for monthly alcohol and tobacco expenditures means that we can detect whether as little as 6% of the $313 monthly income difference between the high-cash and low-cash gift groups is spent on alcohol and tobacco. Our estimated share of the $313 group difference spent on alcohol and tobacco was less than 1%.

How relevant are these results for policy? The BFY cash gifts and the Child Tax Credit provisions of the 2021 American Rescue Plan (ARP) and the Build Back Better (BBB) legislation share some features. For example, in the case of children under age 6, the ARP provided for each child $3,600 annually, paid either monthly or in a lump sum, while in BFY, the annual income difference between the high-cash and low-cash gift groups is $3,720, paid monthly. At the same time, important differences should be noted that limit the generalizability of our findings. ARP payments were allocated per child, so families with more children receive considerably more money than families with fewer children, and ARP payments were made to families with incomes up to $150,000. Moreover, BFY is a poverty reduction intervention specifically designed to begin at the time of the focal child’s birth. The predictable monthly unconditional cash gift is loaded onto a debit card branded as “4MyBaby,” and customer service support in using the card is provided if needed. This combination of features differs somewhat from government payments provided through the tax systems or other mechanisms of direct transfer.

If conditions leading up to or during the pandemic substantially changed mothers’ use of the BFY money, then our study may have limited generalizability beyond the study time frame. We do not have a targeted sample with substance use disorders, nor do we directly measure substance use disorder, so we are unable to test whether substance use or expenditures changed for this subgroup. However, this hypothesis is particularly difficult to test in a general sample like the BFY study because of the low prevalence of substance-use disorder in low-income households [10]. Nonetheless, we did not find that the cash-gift difference between the two groups increased or differentially increased substance use or expenditures for the subset of the BFY sample who reported using any alcohol or cigarettes prior to pregnancy.

Finally, Hawthorne effects, in which participants’ behavior is altered because of their awareness of being observed, are possible, as is the case with any research study. Given the limitations on statistical power inherent in a *n* = 1,000 sample, replications based on larger samples are clearly warranted. We also note that our data on drug use and expenditures come from the self-reports of participants who were aware of their treatment-group status. Because the literature provides mixed evidence on the reliability and validity of these self-reports [32–34], they should be interpreted with caution.

## Conclusions

Our randomized control trial of monthly cash gifts to mothers with newborn infants finds that a cash gift difference of $313 per month did not significantly change maternal use of alcohol, cigarettes, or opioids, or household expenditures on alcohol or cigarettes. Although the structure of our cash gifts differs somewhat from that of a government-provided child allowance, our null effect finding suggests that unconditional cash transfers aimed at families living in poverty are unlikely to induce large changes in substance use and expenditures by recipients. Consistent with most prior findings from other contexts, we find no evidence to support negative stereotypes raised in the policy debate suggesting that substantial portions of a child-allowance-type transfer would be used to purchase alcohol, cigarettes, or opioids.

## Supplementary Information

**Additional file 1.**

## Data Availability

The datasets generated and/or analysed during the current study are available on the Inter-university Consortium for Political and Social Research (ICPSR) repository, https://www.icpsr.umich.edu/web/DSDR/studies/37871/versions/V3. Data for both the baseline and age-1 followup are available at the time of publication.

## Abbreviations

ARP: American Rescue Plan
BBB: Build Back Better
BFY: Baby’s First Years
CES: Consumer Expenditure Survey
EITC: Earned Income Tax Credit

## Footnotes

1 We use the term “mother” throughout the paper to refer to the biological parents who gave birth in the hospitals, regardless of their gender identity. The study did not collect information on gender identity.
