# Supplementary material for: Unconditional cash transfers and maternal substance use: findings from a randomized control trial of low-income mothers with infants in the U.S
Source: BMC Public Health. 2022 May 5;22:897. doi: 10.1186/s12889-022-12989-1 (PMC9070980; doi:10.1186/s12889-022-12989-1)
Supplement: Supplementary file 1 — Additional file 1. [file 12889_2022_12989_MOESM1_ESM.docx]

**SUPPLEMENTAL MATERIALS**

Our Supplemental Materials are organized in two sections. In section A, we provide more information about randomization as well as tables and figures that support the analysis in the text. In section B, we concentrate on comparing the sample interviewed in-person before the onset of the pandemic vs. those interviewed over the phone after the onset of the pandemic. Specifically, we address whether switching from in-person to telephone interviewing in response to pandemic restrictions affects our main results and conclusions.

**Section A: Randomization, Figures and Tables**

The sample’s random assignment mechanism was prepared prior to participant recruitment by University of Michigan’s Survey Research Center (SRC). For each site, or metropolitan area, an excel spreadsheet was created with 100 rows for the high-cash gift group and 150 rows for the low-cash gift group with a unique ID for each row. A random number was generated in Excel, and all the IDs were sorted by the random number, creating a random sequence of group assignment. When a mother in the hospital agreed to participate in the study, staff from SRC would enter in the study consent information, and a program would take the next available ID for that site and present her and the fielding staff with her random ID and group assignment. When hospital IRB approval delayed recruitment in one of our sites (Twin Cities), we increased recruitment goals in the other three sites. To accommodate these increases, we generated additional group IDs using the same 4:6 ratio, randomly sorted, and assigned to mothers upon recruitment using an identical procedure.

**Table S.A1 Regression estimates of baseline differences in pre-treatment substance use**

|  | Pre-Pregnancy | 1^st^ Trimester | 2^nd^ Trimester | 3^rd^ Trimester | Any Trimester | Average cigarettes/drinks per week pre-pregnancy | Average cigarettes/drinks per week during pregnancy |
| --- | --- | --- | --- | --- | --- | --- | --- |
| **A. Smoking** |  |  |  |  |  |  |  |
| Age 1 Sample | 0.004 (0.026) | 0.022 (0.020) | 0.007 (0.018) | -0.002 (0.017) | 0.007 (0.021) | -0.719 (2.673) | -1.538 (1.138) |
| Age 1 Sample, covariate adjusted | 0.002 (0.025) | 0.023 (0.019) | 0.007 (0.017) | -0.002 (0.016) | 0.007 (0.019) | -0.921 (2.418) | -1.543 (0.980) |
| **B. Drinking** |  |  |  |  |  |  |  |
| Age 1 Sample | -0.024 (0.029) | -0.012 (0.011) | -- | -- | -0.026+ (0.014) | -0.474 (0.744) | -0.125 (0.086) |
| Age 1 Sample, covariate adjusted | -0.019 (0.028) | -0.018 (0.013) | -- | -- | -0.031* (0.016) | -0.226 (0.688) | -0.106 (0.069) |
| Logistic regression | X | X | X | X | X |  |  |
| OLS |  |  |  |  |  | X | X |

Notes: The table presents unadjusted and covariate adjusted high-cash and low-cash gift group differences in smoking or drinking before, during, and after pregnancy from different regression models. For each panel, two models were run: a bivariate logistic or linear regression using the age 1 analytic sample and a covariate adjusted logistic or linear regression using the age 1 analytic sample. For logistic regression models, the table presents marginal effect estimates. For example, the estimated proportion of participants having ever smoked in the pre-Pregnancy period is 0.4 percentage points higher in the treatment (high-cash gift) group compared to the low-cash gift group, on average. Covariates include baseline mother and child characteristics: mother's age, completed schooling, household income, net worth, general health, mental health, race and ethnicity, marital status, number of adults in the household, number of other children born to the mother, father living with the mother; child's sex, birth weight, gestational age at birth. For binary outcomes (e.g., ever smoking in 1^st^ trimester), we present the marginal effects of treatment estimated from a logistic regression. Cells containing a dash reflect logistic regression that could not be run due to its low probability and collinearity. Using linear probability model, the covariate adjusted group difference is -0.008 (SE = 0.005, p-value = 0.117) for 2^nd^ Trimester Drinking and is -0.010 (SE = 0.006, p-value = 0.075) for 3^rd^ Trimester Drinking. N= 929 or 930 for the Age 1 analytic sample, depending on the panel. Standard errors are in parentheses.

**Table S.A2 Full regression results for the main impact table**

|  | **Maternal Substance Use Behavior** | | | | **Family Substance Expenditure/Purchase per Week** | | | | | | | |
| --- | --- | --- | --- | --- | --- | --- | --- | --- | --- | --- | --- | --- |
|  | **Pre-pandemic Sample** | | | | **Pre-pandemic Sample** | | | | **Full Sample** | | | |
|  | Alcohol and Cigarette Use Index | Alcohol  Use | Cigarette Use | Opioid  Use | Alcohol and Cigarette (dollars) | Alcohol  (dollars) | Cigarettes (dollars) | Cigarettes  (N packs) | Alcohol and Cigarette (dollars) | Alcohol  (dollars) | Cigarettes (dollars) | Cigarettes  (N packs) |
| Cash-gift treatment effect | 0.057 | 0.031 | 0.028 | -0.031 | 0.050 | 1.174 | -1.123 | -0.144 | 0.394 | 0.668 | -0.296 | -0.039 |
|  | (0.118) | (0.055) | (0.096) | (0.025) | (2.105) | (0.914) | (1.737) | (0.251) | (1.493) | (0.705) | (1.204) | (0.160) |
| MOTHER and HOUSEHOLD | | | | |  |  |  |  |  |  |  |  |
| Age at childbirth (years) | 0.028* | 0.001 | 0.027* | 0.003 | 0.005 | -0.012 | 0.018 | 0.011 | -0.075 | -0.001 | -0.059 | -0.000 |
|  | (0.011) | (0.005) | (0.009) | (0.003) | (0.186) | (0.089) | (0.150) | (0.021) | (0.137) | (0.071) | (0.107) | (0.014) |
| Number of children born to mother | -0.108+ | -0.039 | -0.069 | -0.009 | 0.337 | -0.372 | 0.715 | 0.088 | 0.368 | -0.226 | 0.560 | 0.074 |
|  | (0.056) | (0.024) | (0.047) | (0.015) | (0.884) | (0.360) | (0.727) | (0.102) | (0.616) | (0.265) | (0.505) | (0.068) |
| Health is good or better | 0.165 | 0.119 | 0.044 | -0.114 | 5.794* | 0.052 | 5.728* | 0.894* | 3.909+ | 0.166 | 3.919* | 0.551* |
|  | (0.198) | (0.088) | (0.172) | (0.083) | (2.492) | (1.319) | (2.107) | (0.312) | (2.149) | (1.179) | (1.604) | (0.223) |
| Depression (CESD) | 0.094 | 0.063 | 0.027 | -0.040 | 0.693 | -1.751+ | 2.471 | 0.377 | 0.614 | -1.014 | 1.638 | 0.215 |
|  | (0.139) | (0.063) | (0.122) | (0.035) | (2.653) | (0.918) | (2.395) | (0.366) | (1.819) | (0.812) | (1.540) | (0.208) |
|  | | | | | | | | |  |  |  |  |
| High School Degree or GED | -0.102 | 0.002 | -0.100 | -0.058 | 2.611 | 0.845 | 1.725 | 0.403 | 1.880 | -0.081 | 1.784 | 0.313 |
|  | (0.164) | (0.070) | (0.137) | (0.042) | (2.720) | (1.071) | (2.321) | (0.306) | (1.917) | (0.879) | (1.544) | (0.195) |
| Some College | 0.059 | 0.076 | -0.012 | -0.028 | 1.088 | 0.217 | 0.861 | 0.254 | 1.005 | 0.214 | 0.580 | 0.150 |
|  | (0.193) | (0.082) | (0.166) | (0.058) | (3.122) | (1.386) | (2.695) | (0.381) | (2.206) | (1.169) | (1.799) | (0.237) |
| Associate's Degree | -0.259 | 0.029 | -0.284 | -0.062 | -7.911* | -2.945* | -4.994+ | -0.498 | -4.446+ | -2.006 | -2.680 | -0.206 |
|  | (0.303) | (0.148) | (0.264) | (0.062) | (3.295) | (1.257) | (2.831) | (0.404) | (2.584) | (1.387) | (1.878) | (0.256) |
| Bachelor's Degree | -0.729* | -0.095 | -0.629* | -0.141* | -1.445 | -0.386 | -1.083 | 0.027 | -1.750 | -1.710 | -0.317 | 0.092 |
|  | (0.236) | (0.143) | (0.178) | (0.061) | (4.278) | (2.062) | (3.692) | (0.578) | (2.621) | (1.255) | (2.275) | (0.355) |
| Unknown | -0.541 | -0.177 | -0.357 | 0.974 | -10.491* | -3.951* | -6.478 | -1.028+ | -8.407* | -4.210* | -4.284+ | -0.566 |
|  | (0.429) | (0.369) | (0.219) | (0.828) | (4.545) | (1.551) | (3.986) | (0.574) | (2.755) | (1.302) | (2.464) | (0.362) |
| *Race (omit White)* |  |  |  |  |  |  |  |  |  |  |  |  |
| Black, non-Hispanic | -1.030* | -0.172 | -0.861* | 0.029 | -10.934* | -0.425 | -10.545* | -1.694* | -7.884* | 1.293 | -8.933* | -1.398* |
|  | (0.288) | (0.113) | (0.256) | (0.090) | (4.672) | (1.583) | (4.391) | (0.700) | (3.361) | (1.240) | (3.081) | (0.443) |
| Multi-Race, non-Hispanic | -0.779+ | -0.030 | -0.749* | 0.116 | -5.799 | -0.218 | -5.616 | -1.191 | -4.215 | 2.147 | -6.248 | -1.136+ |
|  | (0.438) | (0.165) | (0.342) | (0.153) | (6.103) | (2.731) | (5.263) | (0.770) | (4.889) | (2.350) | (4.198) | (0.583) |
| Other, non-Hispanic | -0.775+ | -0.063 | -0.713+ | 0.113 | -5.547 | -1.379 | -4.223 | -0.721 | -5.483 | 0.244 | -5.572 | -0.858 |
|  | (0.434) | (0.152) | (0.403) | (0.156) | (7.280) | (2.184) | (6.982) | (1.047) | (5.391) | (1.877) | (5.087) | (0.748) |
| Hispanic | -1.238* | -0.192+ | -1.046* | 0.091 | -11.305* | 0.332 | -11.779* | -1.970* | -9.945* | 0.723 | -10.679* | -1.706* |
|  | (0.275) | (0.113) | (0.242) | (0.101) | (4.471) | (1.665) | (4.322) | (0.690) | (3.232) | (1.225) | (3.076) | (0.450) |
| Unknown | -1.576* | -0.575* | -1.001* | -0.041 | -9.331 | 3.158 | -12.611* | -2.185* | -3.552 | 1.538 | -4.900 | -0.994 |
|  | (0.484) | (0.212) | (0.413) | (0.204) | (7.278) | (2.928) | (6.394) | (0.963) | (4.934) | (2.642) | (5.515) | (0.782) |
|  | | | | | | | | |  |  |  |  |
| Living with partner | -0.101 | -0.139+ | 0.036 | -0.062 | 11.757* | 3.219* | 8.466* | 1.252* | 7.744* | 1.663 | 5.989* | 0.791* |
|  | (0.168) | (0.077) | (0.133) | (0.046) | (3.811) | (1.346) | (3.263) | (0.479) | (2.685) | (1.058) | (2.243) | (0.296) |
| Married | -0.490* | -0.176* | -0.319* | -0.040 | -1.912 | 0.655 | -2.542 | -0.310 | -1.121 | 0.171 | -1.304 | -0.161 |
|  | (0.172) | (0.079) | (0.143) | (0.038) | (2.572) | (1.213) | (2.194) | (0.304) | (1.884) | (0.945) | (1.549) | (0.201) |
| Divorced or Separated | 0.236 | 0.148 | 0.088 | 0.074 | 5.842 | 1.391 | 4.460 | 0.859 | 1.878 | -0.975 | 2.734 | 0.383 |
|  | (0.361) | (0.168) | (0.287) | (0.119) | (7.236) | (2.528) | (6.252) | (0.983) | (4.453) | (1.622) | (3.835) | (0.526) |
| Other | -0.548* | -0.207 | -0.341 | -0.093 | -3.053 | 1.396 | -4.359+ | -0.775* | 0.956 | 0.488 | 0.446 | -0.008 |
|  | (0.265) | (0.130) | (0.218) | (0.073) | (2.985) | (2.129) | (2.522) | (0.392) | (2.518) | (1.769) | (1.901) | (0.285) |
| Unknown | -0.188 | 0.197 | -0.386* | -0.080+ | -8.671* | -4.482* | -4.148 | -0.588 | -6.920* | -4.216* | -2.723 | -0.416+ |
|  | (0.272) | (0.253) | (0.156) | (0.045) | (3.463) | (1.285) | (2.900) | (0.433) | (2.187) | (0.915) | (1.730) | (0.240) |
| Cigarettes per week during pregnancy | 0.027* | 0.002 | 0.025* | 0.004+ | 0.211* | 0.049 | 0.162* | 0.027* | 0.218* | 0.025 | 0.200* | 0.032* |
|  | (0.005) | (0.002) | (0.005) | (0.002) | (0.089) | (0.036) | (0.077) | (0.012) | (0.069) | (0.028) | (0.061) | (0.009) |
| Alcohol drinks per week during pregnancy | 0.194+ | 0.092* | 0.102 | 0.104 | 2.880+ | 1.955 | 0.929 | 0.199 | -0.226 | 0.632 | -0.880* | -0.134+ |
|  | (0.103) | (0.036) | (0.089) | (0.104) | (1.585) | (1.818) | (1.119) | (0.179) | (0.605) | (0.446) | (0.442) | (0.070) |
|  | | | | | | | | |  |  |  |  |
| $10,000 - $14,999 | -0.163 | 0.098 | -0.262 | -0.057 | -5.589 | -1.534 | -4.015 | -0.440 | -4.131 | -0.897 | -3.318 | -0.357 |
|  | (0.190) | (0.078) | (0.159) | (0.048) | (4.306) | (1.244) | (3.860) | (0.583) | (3.002) | (1.008) | (2.642) | (0.343) |
| $15,000 - $19,999 | -0.098 | 0.156+ | -0.256 | -0.055 | -5.975 | 1.230 | -7.243* | -1.012* | -4.185 | 0.515 | -4.780* | -0.578+ |
|  | (0.196) | (0.090) | (0.161) | (0.046) | (3.771) | (1.444) | (3.277) | (0.440) | (2.771) | (1.119) | (2.365) | (0.301) |
| $20,000 - $29,999 | 0.057 | 0.201* | -0.145 | -0.019 | -4.809 | -0.093 | -4.662 | -0.436 | -1.953 | 1.508 | -3.265 | -0.275 |
|  | (0.202) | (0.088) | (0.166) | (0.059) | (4.357) | (1.435) | (3.746) | (0.559) | (3.109) | (1.171) | (2.626) | (0.363) |
| $30,000 or higher | 0.104 | 0.372* | -0.273+ | 0.009 | -7.674+ | 1.543 | -9.213* | -1.227* | -6.135* | 0.712 | -6.873* | -0.827* |
|  | (0.200) | (0.092) | (0.159) | (0.042) | (4.031) | (1.370) | (3.501) | (0.489) | (2.931) | (1.064) | (2.527) | (0.325) |
| Unknown | -0.335 | 0.035 | -0.370* | -0.052 | -9.315* | -0.514 | -8.674* | -1.166* | -5.258+ | 0.636 | -5.889* | -0.704* |
|  | (0.212) | (0.123) | (0.177) | (0.043) | (4.275) | (1.917) | (3.730) | (0.490) | (3.036) | (1.634) | (2.521) | (0.316) |
|  | | | | | | | | |  |  |  |  |
| In debt $1 - $4,999 | -0.154 | -0.179+ | 0.029 | -0.089+ | -0.373 | 0.605 | -0.975 | -0.027 | -1.693 | 0.346 | -1.866 | -0.153 |
|  | (0.193) | (0.096) | (0.152) | (0.052) | (3.418) | (1.411) | (2.844) | (0.438) | (2.649) | (1.141) | (2.181) | (0.305) |
| break even | 0.098 | -0.102 | 0.203 | -0.053 | -1.302 | -0.556 | -0.768 | -0.091 | -1.753 | -0.717 | -1.048 | -0.101 |
|  | (0.170) | (0.082) | (0.133) | (0.047) | (3.180) | (1.043) | (2.750) | (0.394) | (2.157) | (0.815) | (1.855) | (0.240) |
| leftover $1 - $4,999 | -0.011 | -0.083 | 0.075 | -0.049 | 0.178 | 1.294 | -1.095 | -0.180 | -0.986 | 0.904 | -1.830 | -0.229 |
|  | (0.184) | (0.090) | (0.152) | (0.049) | (3.516) | (1.379) | (2.870) | (0.392) | (2.690) | (1.190) | (2.134) | (0.278) |
| leftover $5,000 or more | 0.232 | -0.136 | 0.372* | -0.019 | 1.833 | 1.290 | 0.569 | 0.101 | 2.624 | 2.747+ | 0.379 | 0.094 |
|  | (0.220) | (0.098) | (0.185) | (0.068) | (3.982) | (1.611) | (3.558) | (0.554) | (3.137) | (1.565) | (2.587) | (0.363) |
| Net worth unknown | 0.004 | -0.260* | 0.267 | -0.049 | 4.155 | 2.492 | 1.399 | 0.201 | -0.491 | 0.505 | -1.091 | -0.094 |
|  | (0.207) | (0.105) | (0.181) | (0.052) | (4.603) | (1.803) | (3.799) | (0.516) | (3.086) | (1.236) | (2.549) | (0.338) |
| Number of adults in household | -0.105 | -0.054+ | -0.051 | -0.026+ | 1.531 | 0.751 | 0.776 | 0.112 | 1.270 | 0.483 | 0.780 | 0.134 |
|  | (0.064) | (0.028) | (0.051) | (0.016) | (1.110) | (0.534) | (0.892) | (0.135) | (0.791) | (0.400) | (0.663) | (0.100) |
| Biological father lives in household | 0.204 | 0.033 | 0.174 | 0.012 | 0.201 | -1.679 | 1.860 | 0.170 | 0.575 | -0.753 | 1.192 | 0.089 |
|  | (0.140) | (0.061) | (0.118) | (0.036) | (2.638) | (1.043) | (2.331) | (0.327) | (1.928) | (0.816) | (1.666) | (0.226) |
| **CHILD** |  |  |  |  |  |  |  |  |  |  |  |  |
| Female | -0.070 | -0.051 | -0.018 | 0.023 | 1.790 | 0.517 | 1.335 | 0.273 | 1.172 | 0.020 | 1.287 | 0.199 |
|  | (0.118) | (0.055) | (0.096) | (0.029) | (2.114) | (0.833) | (1.797) | (0.269) | (1.508) | (0.675) | (1.226) | (0.167) |
| Weight at birth (lbs) | -0.098 | -0.084* | -0.013 | -0.003 | -0.476 | -0.166 | -0.299 | -0.037 | -0.859 | -0.190 | -0.603 | -0.093 |
|  | (0.070) | (0.032) | (0.054) | (0.026) | (1.251) | (0.514) | (0.955) | (0.145) | (0.871) | (0.370) | (0.679) | (0.094) |
| Gestational age (weeks) | -0.086 | -0.004 | -0.082+ | 0.002 | -1.553 | -0.376 | -1.183 | -0.178 | -0.735 | -0.046 | -0.693 | -0.085 |
|  | (0.056) | (0.025) | (0.047) | (0.013) | (1.110) | (0.371) | (0.883) | (0.133) | (0.838) | (0.356) | (0.641) | (0.086) |
| Age at age 1 interview | 0.121* | 0.055* | 0.067 | 0.017 | 0.979 | 0.346 | 0.628 | 0.090 | 0.469 | 0.310 | 0.136 | 0.022 |
|  | (0.053) | (0.022) | (0.042) | (0.021) | (0.707) | (0.270) | (0.598) | (0.094) | (0.345) | (0.190) | (0.279) | (0.044) |
|  | | | | | | | | |  |  |  |  |
| missing Cigarettes per week during pregnancy | 1.708 | 0.304 | 1.402 | -0.116 | -1.636 | -1.829 | 0.244 | 0.098 | 17.387 | -2.530* | 19.823 | 2.697 |
|  | (1.133) | (0.276) | (0.876) | (0.082) | (6.386) | (2.190) | (6.516) | (1.078) | (14.836) | (1.247) | (14.687) | (1.894) |
| missing Alcohol drinks per week during pregnancy | 2.800* | 0.855* | 1.946* | -0.117 | -4.522 | 1.594 | -5.936+ | -0.988+ | -1.919 | 0.157 | -1.989 | -0.239 |
|  | (0.293) | (0.281) | (0.382) | (0.124) | (6.293) | (4.187) | (3.467) | (0.543) | (3.754) | (3.529) | (1.851) | (0.290) |
| missing Weight at birth (lbs) | -0.248 | 0.301 | -0.545+ | -0.105 | -7.526 | -1.930 | -5.571 | -0.581 | -3.400 | -2.592 | -0.773 | -0.000 |
|  | (0.398) | (0.189) | (0.316) | (0.129) | (7.811) | (3.003) | (6.630) | (1.026) | (3.213) | (2.403) | (3.611) | (0.443) |
| missing Gestational age (weeks) | -0.693 | 0.095 | -0.791* | -0.044 | -3.035 | 5.051 | -8.052 | -1.311 | -2.132 | 1.898 | -4.124 | -0.589 |
|  | (0.757) | (0.678) | (0.188) | (0.080) | (4.501) | (5.182) | (5.407) | (0.888) | (3.489) | (3.890) | (3.233) | (0.518) |
| **SITE** *(omit Site 1)* |  |  |  |  |  |  |  |  |  |  |  |  |
| Site 2 | 0.235 | 0.017 | 0.217 | -0.016 | 1.591 | 1.630 | -0.027 | -0.594 | 3.283 | 1.661 | 1.496 | -0.187 |
|  | (0.228) | (0.091) | (0.183) | (0.071) | (3.655) | (1.469) | (3.123) | (0.425) | (2.613) | (1.333) | (2.057) | (0.261) |
| Site 3 | 0.149 | 0.117 | 0.027 | -0.063 | -3.969 | -0.270 | -3.775 | -0.555 | -1.545 | -0.151 | -1.463 | -0.167 |
|  | (0.183) | (0.084) | (0.147) | (0.051) | (2.773) | (1.143) | (2.308) | (0.374) | (1.971) | (0.970) | (1.590) | (0.231) |
| Site 4 | 0.110 | 0.148+ | -0.040 | -0.106* | -0.379 | 0.369 | -0.676 | -0.476 | 1.344 | 0.647 | 0.657 | -0.123 |
|  | (0.185) | (0.086) | (0.148) | (0.044) | (2.923) | (1.358) | (2.342) | (0.335) | (2.079) | (1.080) | (1.573) | (0.206) |
| Interview via phone | -0.319 | -0.243 | -0.077 | -0.272 | -11.451 | -0.260 | -11.148 | -1.304 | -4.396* | -0.125 | -4.089* | -0.514* |
|  | (0.553) | (0.245) | (0.449) | (0.232) | (9.267) | (3.529) | (7.951) | (1.216) | (1.384) | (0.717) | (1.082) | (0.148) |
| Constant | 3.989+ | 0.599 | 3.360+ | 0.041 | 63.163 | 13.247 | 50.161 | 7.306 | 41.650 | 0.482 | 40.635+ | 5.061 |
|  | (2.146) | (0.960) | (1.775) | (0.493) | (40.701) | (13.966) | (32.634) | (4.899) | (30.722) | (12.900) | (23.897) | (3.184) |
| N | 597 | 597 | 598 | 597 | 593 | 595 | 595 | 595 | 920 | 922 | 927 | 927 |
| R-sq | 0.321 | 0.145 | 0.349 | 0.143 | 0.148 | 0.092 | 0.155 | 0.171 | 0.133 | 0.052 | 0.160 | 0.187 |
| Note: This table presents the full set of coefficients and standard errors for the treatment indicator and all the control variables from the regression estimates reported in Table 1. Treatment indicates assignment into the high-cash group ($333 monthly payments) as opposed to the low-cash group ($20 monthly payments). The pre-pandemic panel report treatment effects for the sample interviewed prior to the onset of the pandemic. The pre-pandemic sample is missing substance use measures because these sensitive items were part of an audio computer-assisted survey which was administered during an in-home visit prior to the onset of the pandemic and were dropped during the pandemic when survey switched interview format from in-person to phone interviews. Alcohol use, cigarette use, and opioid use are each measured from a maternal report of the frequency of substance use since the baby’s birth (i.e., the last year) on a 5-point response scale (0: never in last year; 1: less than 1 time per month; 2: several times per month; 3: several times per week; 4: everyday). The Alcohol and Cigarette Use Index, a pre-registered outcome, is an additive index of the two measures, alcohol use and cigarette use, which range from 0 to 8. The average week refers to the average week in the most recent month prior to Age 1 interview. The dollar expenditure on cigarettes per week is estimated by multiplying reported packs of cigarettes with the average cost of a cigarette pack by site. According to the Centers for Disease Control and Prevention, the average cost of a pack of cigarette in 2019 for the four states in BFY were: LA -$6.08, MN -$9.13, NE-$5.78, NY-$10.53 Alcohol and cigarette expenditure outcomes were winsorized at the 99^th^ percentile to adjust for extreme values. CESD is Center for Epidemiological Studies – Depression Scale. 1 participant with substance use measures included in the pre-pandemic panel started the interview in person but completed the interview over the phone. Effect sizes on substance use outcomes are identical if we exclude this participant. 8 participants who were interviewed prior to the onset of the pandemic and have valid substance expenditure data were excluded from panel A because they were missing substance use measures. Including the 8 participants decreases the effect size on cigarette purchased and alcohol expenditure to 0.004 and 0.011, respectively. Standard errors are in parentheses. + p<0.10, * p<0.05 | | | | | | | | | | | | |

**Table S.A3 Impacts on continuous measures of substance use**

|  | Smoking Occasions per week | Drinking Occasions per week | Number of Drinks per week | Opioid Use Occasions per week |
| --- | --- | --- | --- | --- |
| Low-cash gift group mean (standard deviation) | 0.879 (2.133) | 0.100 (0.406) | 0.199 (0.622) | 0.086 (0.707) |
| Cash-gift treatment effect (standard error) | 0.038 (0.151) | 0.019 (0.031) | 0.035 (0.058) | -0.055+ (0.029) |
| Effect size | 0.018 | 0.047 | 0.056 | -0.078 |
| *p*-value, unadjusted | 0.802 | 0.544 | 0.549 | 0.058 |
| *p*-value, adjusted | 0.866 | 0.866 | 0.866 | 0.283 |
| N | 598 | 597 | 597 | 597 |

Notes: Alcohol use, cigarette use, and opioid use are each measured from a maternal report of the frequency of substance use since the baby’s birth (i.e., the last year) on a 5-point response scale (0: never in last year; 1: less than 1 time per month; 2: several times per month; 3: several times per week; 4: everyday). We convert these ordinal measures into continuous measures of substance use occasions per week using the following rules. 7 occasions per week when mothers report drinking daily; 3.5 when mothers report drinking several times per week, or (1/2)*(1+6) which is the midpoint or average of drinking 1 time per week and 6 times per week; 0.615 when mothers drink several times per month, or (1/2)*((12+52)/52) which is the average of drinking 1 time per week and 1 time per month; 0.115 when mothers drink less than 1 time per month, or (1/2)*((1+11)/52) which is the average of drinking 1 time per year and 11 time per year. Point estimates were not sensitive to lower or higher bound conversion assumptions (e.g., converting several times per week to 2 or 5 times per week). To create the estimated number of drinks per week, we take the product of the estimated number of occasions per week and the typical number of drinks per occasion reported. Estimated treatment effect controls for baby’s age (in months) at time of interview, whether the interview was conducted over the phone, and the following pre-registered baseline mother and child characteristics: mother’s age, completed schooling, household income, net worth, general health, mental health, race and ethnicity, marital status, number of adults in the household, number of other children born to the mother, number of cigarettes per week during pregnancy, number of drinks per week during pregnancy, father living with the mother; child’s sex, birth weight, gestational age at birth. Effect size is computed by dividing the treatment effect with the standard deviation of the low-cash gift group. Unadjusted p-values and pre-registered Westfall and Young adjusted p-values, which adjust for multiple hypothesis testing, are both reported. For the Westfall-Young adjustment, the four substance use measures are put into one family. The sample of 597 or 598 in this table is smaller than the full age 1 analytic sample of this sample. This is because these sensitive substance use items were part of an audio computer-assisted survey which was administered during an in-home visit prior to the onset of the pandemic and were dropped during the pandemic when survey switched interview format from in-person to phone interviews. Standard errors are in parentheses. + p<0.10; * p<0.05; ** p<0.01.

**Table S.A4 Impact estimates on maternal substance use from ordered logits**

|  | Alcohol  Use | Cigarette  Use | Opioid  Use |
| --- | --- | --- | --- |
| Cash-gift treatment effect, odds ratio (standard error) | 1.107  (0.211) | 1.063  (0.271) | 0.971  (0.572) |
| *p*-value | 0.592 | 0.809 | 0.96 |
| N Analysis | 597 | 598 | 597 |
| N Low-cash gift group | 339 | 340 | 340 |
| % Never in the last year | 60.18 | 79.41 | 96.47 |
| % Less than one time per month | 32.74 | 2.06 | 1.76 |
| % Several times per month | 6.78 | 3.53 | 0.29 |
| % Several times per week | 0.00 | 5.59 | 0.59 |
| % Every day | 0.29 | 9.41 | 0.88 |

Notes: Table presents treatment effect in odds ratio from an ordered logit. Alcohol use, cigarette use, and opioid use are each measured from a maternal report of the frequency of substance use since the baby’s birth (i.e., the last year) on a 5-point response scale (0: never in last year; 1: less than 1 time per month; 2: several times per month; 3: several times per week; 4: everyday). Estimated treatment effect controls for baby’s age (in months) at time of interview, whether the interview was conducted over the phone, and the following pre-registered baseline mother and child characteristics: mother’s age, completed schooling, household income, net worth, general health, mental health, race and ethnicity, marital status, number of adults in the household, number of other children born to the mother, number of cigarettes per week during pregnancy, number of drinks per week during pregnancy, father living with the mother; child’s sex, birth weight, gestational age at birth. Effect size is computed by dividing the treatment effect with the standard deviation of the low-cash gift group. Unadjusted p-values and pre-registered Westfall and Young adjusted p-values, which adjust for multiple hypothesis testing, are both reported. For the Westfall-Young adjustment, the three substance use measures are placed into one family. The sample of 597 or 598 in this table is smaller than the full age 1 analytic sample of this sample. This is because these sensitive substance use items were part of an audio computer-assisted survey which was administered during an in-home visit prior to the onset of the pandemic and were dropped during the pandemic when survey switched interview format from in-person to phone interviews. Standard errors are in parentheses. + p<0.10; * p<0.05; ** p<0.01.

**Table S.A5 Subgroup analysis of treatment effects by prior alcohol use during pregnancy or 3-month before pregnancy**

|  | A. Prior Alcohol Use | | | | B. No Prior Alcohol Use | | | | C. Testing equivalence of two treatment coefficients from the subgroups, unadjusted p-value | | | |
| --- | --- | --- | --- | --- | --- | --- | --- | --- | --- | --- | --- | --- |
|  | 1 | 2 | 3 | 4 | 1 | 2 | 3 | 4 | 1 | 2 | 3 | 4 |
|  | Maternal Alcohol and Cigarette Use Index | Maternal Opioid Use | Cigarettes purchased for family in average week  (N packs) | Alcohol expenditure for family in average week (dollars) | Maternal Alcohol and Cigarette Use Index | Maternal Opioid Use | Cigarettes purchased for family in average week  (N packs) | Alcohol expenditure for family in average week (dollars) | Maternal Alcohol and Cigarette Use Index | Maternal Opioid Use | Cigarettes purchased for family in average week  (N packs) | Alcohol expenditure for family in average week (dollars) |
| Low-cash gift group mean | 1.812 | 0.070 | 1.299 | 6.463 | 0.878 | 0.079 | 0.791 | 2.346 | <0.001 | 0.878 | .047 | <0.001 |
| Cash-gift treatment effect (SE) | 0.510  (0.323) | -0.014  (0.056) | -0.040  (0.426) | -2.207  (1.923) | -0.014  (0.128) | -0.039  (0.030) | -0.060  (0.194) | 1.544+  (0.802) | 0.071 | 0.857 | 0.895 | 0.029 |
| Effect size | 0.319 | -0.030 | -0.013 | -0.238 | -0.009 | -0.083 | -0.019 | 0.167 |  |  |  |  |
| *p*-value, unadjusted | 0.118 | 0.808 | 0.925 | 0.253 | 0.915 | 0.188 | 0.756 | 0.055 |  |  |  |  |
| *p*-value, adjusted | 0.225 | 0.836 | 0.928 | 0.437 | 0.922 | 0.378 | 0.742 | 0.103 |  |  |  |  |
| N | 144 | 144 | 232 | 229 | 453 | 453 | 694 | 692 |  |  |  |  |

Notes: Alcohol use, cigarette use, and opioid use are each measured from a maternal report of the frequency of substance use since the baby’s birth (i.e., the last year) on a 5-point response scale (0: never in last year; 1: less than 1 time per month; 2: several times per month; 3: several times per week; 4: everyday). The Alcohol and Cigarette Use Index, a pre-registered outcome, is an additive index of the two measures, alcohol use and cigarette use, which range from 0 to 8. The average week refers to the average week in the most recent month prior to Age 1 interview. Alcohol and cigarette expenditure outcomes were winsorized at the 99^th^ percentile to adjust for extreme values. Estimated treatment effect controls for baby’s age (in months) at time of interview, whether the interview was conducted over the phone, and the following pre-registered baseline mother and child characteristics: mother’s age, completed schooling, household income, net worth, general health, mental health, race and ethnicity, marital status, number of adults in the household, number of other children born to the mother, number of cigarettes per week during pregnancy, number of drinks per week during pregnancy, father living with the mother; child’s sex, birth weight, gestational age at birth. Effect size is computed by dividing the treatment effect with the standard deviation of the full age 1 analytic samples low-cash gift group. Unadjusted p-values and pre-registered Westfall and Young adjusted p-values, which adjust for multiple hypothesis testing, are both reported. For the Westfall-Young adjustment, the two substance use measures are placed into one family and the two expenditure measures are placed into one family. Sample size for the two substance use measures are smaller than the sample size for the two expenditure measures because the substance use items were part of an audio computer-assisted survey for sensitive items, which was administered during an in-home visit prior to the onset of the pandemic and were dropped during the pandemic when survey switched interview format from in-person to phone interviews. Panels A and B respectively report on the subgroup of participants who reported at baseline data collection any alcohol drinking during pregnancy or 3-months before pregnancy. Panel C reports the unadjusted *p*-values that test the difference in the corresponding coefficients between panel A and B. Standard errors are in parentheses. The adjusted *p*-value of the interaction of treatment effect on alcohol expenditure and prior alcohol use indicator (column 4 of panel C) is 0.086. + p<0.10; * p<0.05; ** p<0.01.

**Table S.A6 Subgroup analysis of treatment effects by prior smoking during pregnancy or 3-month before pregnancy**

|  | A. Prior Smoking | | | | B. No Prior Smoking | | | | C. Testing equivalence of two treatment coefficients from the subsamples, unadjusted p-value | | | |
| --- | --- | --- | --- | --- | --- | --- | --- | --- | --- | --- | --- | --- |
|  | 1 | 2 | 3 | 4 | 1 | 2 | 3 | 4 | 1 | 2 | 3 | 4 |
|  | Maternal Alcohol and Cigarette Use Index | Maternal Opioid Use | Cigarettes purchased for family in average week  (N packs) | Alcohol expenditure for family in average week (dollars) | Maternal Alcohol and Cigarette Use Index | Maternal Opioid Use | Cigarettes purchased for family in average week  (N packs) | Alcohol expenditure for family in average week (dollars) | Maternal Alcohol and Cigarette Use Index | Maternal Opioid Use | Cigarettes purchased for family in average week  (N packs) | Alcohol expenditure for family in average week (dollars) |
| Low-cash gift group mean | 3.178 | 0.137 | 2.898 | 4.817 | 0.545 | 0.060 | 0.437 | 3.088 | <0.001 | 0.212 | <0.001 | 0.084 |
| Cash-gift treatment effect (SE) | -0.165  (0.416) | -0.005  (0.074) | 0.729  (1.043) | -0.271  (2.334) | -0.004  (0.083) | -0.046+  (0.026) | -0.166  (0.109) | 0.586  (0.774) | 0.789 | 0.273 | 0.750 | 0.788 |
| Effect size | -0.103 | -0.011 | 0.237 | -0.022 | -0.003 | -0.098 | -0.054 | 0.063 |  |  |  |  |
| *p*-value, unadjusted | 0.693 | 0.948 | 0.486 | 0.908 | 0.964 | 0.079 | 0.127 | 0.449 |  |  |  |  |
| *p*-value, adjusted | 0.917 | 0.956 | 0.736 | 0.917 | 0.967 | 0.225 | 0.226 | 0.453 |  |  |  |  |
| N | 126 | 126 | 185 | 184 | 471 | 471 | 742 | 738 |  |  |  |  |

Notes: Alcohol use, cigarette use, and opioid use are each measured from a maternal report of the frequency of substance use since the baby’s birth (i.e., the last year) on a 5-point response scale (0: never in last year; 1: less than 1 time per month; 2: several times per month; 3: several times per week; 4: everyday). The Alcohol and Cigarette Use Index, a pre-registered outcome, is an additive index of the two measures, alcohol use and cigarette use, which range from 0 to 8. The average week refers to the average week in the most recent month prior to Age-1 interview. Alcohol and cigarette expenditure outcomes were winsorized at the 99^th^ percentile to adjust for extreme values. Estimated treatment effect controls for baby’s age (in months) at time of interview, whether the interview was conducted over the phone, and the following pre-registered baseline mother and child characteristics: mother’s age, completed schooling, household income, net worth, general health, mental health, race and ethnicity, marital status, number of adults in the household, number of other children born to the mother, number of cigarettes per week during pregnancy, number of drinks per week during pregnancy, father living with the mother; child’s sex, birth weight, gestational age at birth. Effect size is computed by dividing the treatment effect with the standard deviation of the full age-1 analytic samples low-cash gift group. Unadjusted p-values and pre-registered Westfall and Young adjusted p-values, which adjust for multiple hypothesis testing, are both reported. For the Westfall-Young adjustment, the two substance use measures are placed into one family and the two expenditure measures are placed into one family. Sample size for the two substance use measures are smaller than the sample size for the two expenditure measures because the substance use items were part of an audio computer-assisted survey for sensitive items, which was administered during an in-home visit prior to the onset of the pandemic and were dropped during the pandemic when survey switched interview format from in-person to phone interviews. Panels A and B respectively report on the subgroup of participants who reported at baseline data collection any smoking during pregnancy or 3-months before pregnancy. Panel C reports the unadjusted *p*-values that test the difference in the corresponding coefficients between panel A and B. Standard errors are in parentheses. + p<0.10; * p<0.05; ** p<0.01.

**Section B. Pre- and post-pandemic-onset samples**

A potential limitation of the study is the occurrence of the COVID-19 pandemic when BFY’s age-1 follow-up data collection was about two-thirds complete. Because both the treatment (high-cash gift) and control (low-cash gift) groups were affected by the pandemic and lockdown, the internal validity of BFY causal impact estimates is largely intact. However, the results from the full analytic sample are only generalizable to the conditions that prevailed before and after the onset of the pandemic. Data from the Consumer Expenditure Surveys shows that while cigarette spending among households in the bottom income quintile changed relatively little between 2019 and 2020 (falling from $7.87 billion to $7.39 billion), spending on alcohol fell substantially more (from $5.51 billion to $3.,96 billion.)^[[1]](#endnote-2)^

Because of the COVID-19 pandemic, BFY stopped all in-home data collection on March 13, 2020 and, two days later, began administering telephone interviews. By the end of the age-1 fielding period, 597 of the age-1 interviews with valid substance use data had been conducted in person and the rest by phone^[[2]](#endnote-3)^. As described in the main text, the more sensitive questions in the survey (including those about maternal tobacco, alcohol and opioid use) were asked using pre-recorded audio played over headphones (audio-CASI) rather than directly by the interviewer. As this procedure was not possible over the phone, the substance use measures are available only for the subsample interviewed in person prior to the onset of the pandemic. This created a problem of missing data for estimating treatment effects on substance use measures. Furthermore, the pre-pandemic sample may have been affected by the somewhat differential response rates between high- and low-cash gift groups that prevailed before the pandemic began. Both concerns raise questions about the robustness of the pre-pandemic sample analysis of substance use. As seen in Table 1 of the main text, we were able to estimate treatment impacts on substance expenditure measures for both the pre-pandemic sample and the full age-1 analytic sample. Our treatment effect estimates did not appear sensitive to pandemic-induced sample selection. The treatment effect estimates on substance expenditure using the pre-pandemic sample and the full age-1 analytic sample were similar. Nonetheless, we address the concern of missing data and sample selection more closely through multiple tests and weighting schemes in this section of the supplemental materials.

The pre-pandemic subsample has largely balanced treatment and control groups (i.e., high- and low-cash groups) and appears no different from the subsample interviewed following the onset of the pandemic. We tested the overall baseline balance between high- and low-cash gift groups with a joint test of orthogonality using a probit model with robust standard errors and site-level fixed effects. That is, we tested the null hypothesis of whether all of the coefficients of the baseline characteristic variables predicting treatment status were equal to zero and thus orthogonal to treatment status. We were not able to reject the null hypothesis of no group difference at the .05 alpha level (*p*-value = 0.075). We then tested the null hypothesis of no group difference between the pre-pandemic and post-pandemic-onset subsamples and again were not able the reject the null hypothesis (*p*-value = 0.636). In tables S.B1 and S.B2, we present the full set of individual baseline equivalence tests for the two comparisons. While we did not see statistically significant differences in the overall balance of the pre-pandemic sample, the race composition and baseline health between the high- and low-cash gift groups appeared to be different, such that a higher proportion of mothers in the treatment group identified as Black and reported their health is good or better.

To further address potential group differences that may result from missing data or sample selection, we applied two types of weights to the pre-pandemic sample and full age-1 sample. We used a machine learning algorithm package TWANG^[[3]](#endnote-4)^ (Toolkit for Weighting and Analysis of Nonequivalent Groups) which uses generalized boosted models to flexibly generate conditional probabilities, or propensity scores, and relevant weights for analysis. The key innovation of TWANG is that the algorithm is optimized to create weights that minimize group differences. First, we adjusted for non-response through inverse probability weighting^[[4]](#endnote-5)^—the inverse of the probability of participating in pre-pandemic age 1 data collection to make the pre-pandemic sample have characteristics similar to the full BFY study sample (N=1,000). The non-response weighting aims to effectively recreate the treatment and control group balance at baseline. We created a second set of weights that are typically used to estimate the average treatment effect on the treated (ATT). The control group participants are weighted by the probability of being in the treatment group to make the control group participants in the pre-pandemic sample have similar characteristics as the treatment group in the pre-pandemic sample, directly minimizing group differences. We repeat this process with the full age-1 analytic sample to adjust for any group differences that may have emerged from the small attrition between baseline and age-1 follow-up. We run our main impact analysis using both weights (see Tables S.B3 and S.B4). All of the null effect findings are robust to both weighting adjustments.

In sum, the pre-pandemic sample appears balanced and similar to the full age 1 analytic sample; weighed analyses that remove differences between the treatment and control groups do not change any of the main findings.

**Table S.B1 Baseline Balance of the Age 1 Pre-pandemic Analytic Sample (N = 598)**

|  | Low-Cash Gift | | High-Cash Gift | | Std Mean Difference | | | | |
| --- | --- | --- | --- | --- | --- | --- | --- | --- | --- |
|  | Mean (sd) | N | Mean (sd) | N | Hedges’ g | Cox’s Index | | p-value | |
| **CHILD** |  |  |  |  |  |  | |  | |
| Female | 0.509 | 340 | 0.442 | 258 |  | -0.163 | | 0.099 | |
| Weight at birth (lbs) | 7.076  (1.049) | 339 | 7.175  (1.021) | 258 | 0.096 |  | | 0.184 | |
|  |  |  |  |  |  |  | |  | |
| Gestational age (weeks) | 39.025 | 337 | 39.052 | 258 | 0.021 |  | | 0.682 | |
|  | (1.309) |  | (1.290) |  |  |  | |  | |
| **MOTHER** |  |  |  |  |  |  | |  | |
| Age at childbirth (years) | 26.697 | 340 | 27.453 | 258 | 0.130 |  | | 0.082 | |
|  | (5.784) |  | (5.869) |  |  |  | |  | |
| Education (years) | 11.914 | 336 | 11.953 | 257 | 0.013 |  | | 0.872 | |
|  | (2.955) |  | (3.029) |  |  |  | |  | |
| Mother race/ethnicity: |  |  |  |  |  |  | |  | |
| White, non-Hispanic | 0.124 | 340 | 0.058 | 258 |  | -0.504 | | 0.005 | |
| Black, non-Hispanic | 0.388 | 340 | 0.481 | 258 |  | 0.230 | | 0.033 | |
| Multiple, non-Hispanic | 0.053 | 340 | 0.035 | 258 |  | -0.263 | | 0.282 | |
| other or unknown | 0.044 | 340 | 0.027 | 258 |  | -0.306 | | 0.287 | |
| Hispanic | 0.391 | 340 | 0.399 | 258 |  | 0.020 | | 0.533 | |
| Mother marital status: |  |  |  |  |  |  | |  | |
| never married | 0.438 | 340 | 0.527 | 258 |  | 0.216 | | 0.050 | |
| single, living with partner | 0.265 | 340 | 0.213 | 258 |  | -0.174 | | 0.179 | |
| Married | 0.206 | 340 | 0.202 | 258 |  | -0.015 | | 0.953 | |
| divorced/separated | 0.047 | 340 | 0.019 | 258 |  | -0.566 | | 0.065 | |
| other or unknown | 0.044 | 340 | 0.039 | 258 |  | -0.076 | | 0.720 | |
| Health is good or better | 0.874 | 340 | 0.930 | 258 |  | 0.393 | | 0.022 | |
| Depression (CESD) | 0.689 | 340 | 0.671 | 258 | -0.041 |  | | 0.528 | |
|  | (0.437) |  | (0.430) |  |  |  | |  | |
| Cigarettes per week during pregnancy | 5.302 | 338 | 3.417 | 257 | -0.104 |  | | 0.191 | |
|  | (21.764) |  | (11.835) |  |  |  | |  | |
| Alcohol drinks per week during pregnancy | 0.044 | 338 | 0.039 | 258 | -0.010 |  | | 0.904 | |
|  | (0.499) |  | (0.473) |  |  |  | |  | |
| Number of children born to mother | 2.388 | 340 | 2.547 | 258 | 0.117 |  | | 0.139 | |
|  | (1.300) |  | (1.428) |  |  |  | |  | |
| Number of adults in household | 2.132 | 340 | 1.992 | 258 | -0.141 |  | | 0.072 | |
|  | (1.029) |  | (0.942) |  |  |  | |  | |
| Biological father lives in household | 0.394 | 340 | 0.329 | 258 |  | -0.171 | | 0.121 | |
| Household combined income | 22,891.713 | 321 | 19,983.741 | 240 | -0.159 |  | | 0.060 | |
|  | (20,613.904) |  | (14,434.832) |  |  |  | |  | |
| Household income unknown | 0.056 | 340 | 0.070 | 258 |  | 0.144 | | 0.516 | |
| Household net worth | -1,855.284 | 306 | -2,182.517 | 232 | -0.012 |  | | 0.894 | |
|  | (35,803.149) |  | (13,190.926) |  |  |  | |  | |
| Household net worth unknown | 0.100 | 340 | 0.101 | 258 |  | 0.007 | | 0.982 | |
| Joint test: Chi^2^(30)= 37.02, p-value= 0.075, n=592. | | | | | | |  | |  |

Notes: *P*-values were derived from a series of OLS bivariate regressions in which each respective baseline characteristic was regressed on the treatment status indicator using robust standard errors and site-level fixed effects. The joint test of orthogonality was conducted using a probit model with robust standard errors and site-level fixed effects. Standardized mean differences were calculated using Hedges’ g for continuous variables and Cox’s Index for dichotomous variables. If there were more than 10 missing cases for a covariate, missing data dummies were included in the table and the joint test. If there were less than 10 cases missing, missing data dummies were not included in the table but were included in the joint test. All respondents with missing data on gestational age are in the control group. Thus, this dummy was removed from the joint test due to perfectly predicting failure. This results in a slightly smaller sample for the joint test. Chi-square tests of independence were conducted for the two categorical variables: mother race/ethnicity and mother marital status. For race, *p* = 0.019; and for marital status, *p*>0.05.

**Table S.B2 Baseline Balance between Pre-pandemic sample and post-pandemic-onset sample (N = 930)**

|  | Pre-Pandemic | | Post-Pandemic Onset | | Std Mean Difference | | |
| --- | --- | --- | --- | --- | --- | --- | --- |
|  | Mean (sd) | N | Mean (sd) | N | Hedges’ g | Cox’s Index | *p*-value |
| **CHILD** |  |  |  |  |  |  |  |
| Female | 0.481 | 605 | 0.520 | 325 |  | 0.095 | 0.254 |
| Weight at birth(lbs) | 7.114 | 604 | 7.127 | 324 | 0.013 |  | 0.884 |
|  | (1.034) |  | (1.100) |  |  |  |  |
| Gestational age(weeks) | 39.029 | 602 | 39.138 | 324 | 0.088 |  | 0.200 |
|  | (1.297) |  | (1.129) |  |  |  |  |
| **MOTHER** |  |  |  |  |  |  |  |
| Age at birth (years) | 27.063 | 605 | 27.252 | 325 | 0.033 |  | 0.858 |
|  | (5.826) |  | (5.782) |  |  |  |  |
| Education(years) | 11.932 | 600 | 11.798 | 321 | -0.046 |  | 0.421 |
|  | (2.982) |  | (2.708) |  |  |  |  |
| Race/Ethnicity |  |  |  |  |  |  |  |
| White, non-Hispanic | 0.096 | 605 | 0.095 | 325 |  | -0.007 | 0.681 |
| Black, non-Hispanic | 0.430 | 605 | 0.372 | 325 |  | -0.146 | 0.189 |
| multiple, non-Hispanic | 0.043 | 605 | 0.028 | 325 |  | -0.269 | 0.317 |
| Other or unknown | 0.038 | 605 | 0.031 | 325 |  | -0.128 | 0.752 |
| Hispanic | 0.393 | 605 | 0.474 | 325 |  | 0.200 | 0.101 |
| Marital status |  |  |  |  |  |  |  |
| Never married | 0.476 | 605 | 0.403 | 325 |  | -0.180 | 0.046 |
| single, living with partner | 0.245 | 605 | 0.252 | 325 |  | 0.023 | 0.857 |
| Married | 0.203 | 605 | 0.234 | 325 |  | 0.110 | 0.368 |
| divorced/separated | 0.035 | 605 | 0.046 | 325 |  | 0.172 | 0.488 |
| Other or unknown | 0.041 | 605 | 0.065 | 325 |  | 0.294 | 0.105 |
| Health is good or better | 0.899 | 605 | 0.895 | 325 |  | -0.026 | 0.902 |
| Mother depression (CESD) | 0.677 | 605 | 0.677 | 325 | -0.001 |  | 0.770 |
|  | (0.429) |  | (0.474) |  |  |  |  |
| Cigarettes per week during pregnancy | 4.436 | 602 | 3.288 | 321 | -0.067 |  | 0.499 |
|  | (18.065) |  | (15.303) |  |  |  |  |
| Alcohol drinks per week during pregnancy | 0.041 | 603 | 0.210 | 324 | 0.130 |  | 0.158 |
|  | (0.485) |  | (2.096) |  |  |  |  |
| Number of children born to mother | 2.464 | 605 | 2.465 | 325 | 0.000 |  | 0.796 |
|  | (1.357) |  | (1.454) |  |  |  |  |
| Number of adults in household | 2.063 | 605 | 2.052 | 325 | -0.011 |  | 0.803 |
|  | (0.993) |  | (0.950) |  |  |  |  |
| Biological father lives in household | 0.365 | 605 | 0.425 | 325 |  | 0.152 | 0.101 |
| Household combined income | 21,550.950 | 566 | 22,174.624 | 303 | 0.032 |  | 0.565 |
|  | (18,250.018) |  | (21177.855) |  |  |  |  |
| Household income unknown | 0.064 | 605 | 0.068 | 325 |  | 0.039 | 0.836 |
| Household net worth | -2,034.372 | 540 | -3,741.581 | 291 | -0.065 |  | 0.309 |
|  | (28,306.182) |  | (21,582.030) |  |  |  |  |
| Household net worth unknown | 0.107 | 605 | 0.105 | 325 |  | -0.013 | 0.765 |
| Joint test: Chi^2^(30)= 25.795, *p*-value= 0.636, n=930. | | | | | | | |

Notes: P-values were derived from a series of ordinary lease squares bivariate regressions in which each respective baseline characteristic was regressed on an indicator for whether the participant was interviewed prior to the onset of the pandemic in person, or after the onset of the pandemic by phone using robust standard errors and site-level fixed effects. The joint test of orthogonality was conducted using a probit model with robust standard errors and site-level fixed effects. Standardized mean differences were calculated using Hedges’ g for continuous variables and Cox’s Index for dichotomous variables. If there were more than 10 missing cases for a covariate, missing data dummies were included in the table and the joint test. If there were less than 10 cases missing, missing data dummies were not included in the table but were included in the joint test. All respondents with missing data on gestational age are in the low-cash gift group. Thus, this dummy was removed from the joint test due to perfectly predicting failure. This results in a slightly smaller sample for the joint test. Chi-square tests of independence were conducted for the two categorical variables: mother race/ethnicity and mother marital status. For both tests, p>0.05. The number of pre-pandemic sample of 605 is larger than the 597 reported pre-pandemic panel of the main impact table because 8 participants in the pre-pandemic were missing the substance use outcome measures. The baseline equivalence is not sensitive to the exclusion of these 8 participants. The joint test of orthogonality yields a chi-square value of 25.074 (*p*-value = 0.674), and all the individual bivariate regressions fail to reject the same null hypothesis tests as presented in this table.

**Table S.B3 Treatment Effect on Substance Use and Expenditure by Age 1 with Non-Response Weights**

|  | **Maternal Substance Use Behavior** | | | | **Family Substance Expenditure/Purchase per Week** | | | |
| --- | --- | --- | --- | --- | --- | --- | --- | --- |
|  | Alcohol and Cigarette Use Index | Alcohol  Use | Cigarette Use | Opioid  Use | Alcohol and Cigarette (dollars) | Alcohol  (dollars) | Cigarettes (dollars) | Cigarettes  (N packs) |
| ***A. Pre-pandemic Sample*** |  |  |  |  |  |  |  |  |
| Low-cash gift group mean | 1.107 | 0.476 | 0.629 | 0.075 | 12.495 | 3.446 | 9.003 | 1.291 |
| Cash-gift treatment effect (SE) | 0.057  (0.118) | 0.026  (0.056) | 0.032  (0.096) | -0.031  (0.025) | -0.079  (2.106) | 1.145  (0.915) | -1.223  (1.736) | -0.156  (0.251) |
| Effect size | 0.036 | 0.041 | 0.024 | -0.066 | -0.003 | 0.123 | -0.053 | -0.047 |
| *p*-value, unadjusted | 0.632 | 0.635 | 0.742 | 0.219 | 0.970 | 0.212 | 0.481 | 0.533 |
| *p*-value, adjusted | 0.870 | 0.870 | 0.870 | 0.563 | 0.970 | 0.420 | 0.658 | 0.683 |
| Unweighted N | 597 | 597 | 598 | 597 | 593 | 595 | 595 | 595 |
| ***B. Full Sample*** |  |  |  |  |  |  |  |  |
| Low-cash gift group mean | 1.107 | 0.476 | 0.629 | 0.075 | 10.045 | 3.449 | 6.611 | 0.929 |
| Cash-gift treatment effect (SE) | 0.057  (0.118) | 0.026  (0.056) | 0.032  (0.096) | -0.031  (0.025) | 0.339  (1.496) | 0.680  (0.708) | -0.362  (1.203) | -0.046  (0.160) |
| Effect size | 0.036 | 0.04 | 0.024 | -0.066 | 0.015 | 0.073 | -0.019 | -0.018 |
| *p*-value, unadjusted | 0.632 | 0.637 | 0.741 | 0.219 | 0.821 | 0.337 | 0.764 | 0.772 |
| *p*-value, adjusted | 0.864 | 0.864 | 0.864 | 0.580 | 0.938 | 0.625 | 0.938 | 0.939 |
| Unweighted N | 597 | 597 | 598 | 597 | 920 | 922 | 927 | 927 |

Notes: Non-response weights were created to weight the pre-pandemic sample and as well as the full age-1 analytic sample to look like the full BFY study sample (N= 1,000; T=400, C=600). Alcohol use, cigarette use, and opioid use are each measured from a maternal report of the frequency of substance use since the baby’s birth (i.e., the last year) on a 5-point response scale (0: never in last year; 1: less than 1 time per month; 2: several times per month; 3: several times per week; 4: everyday). The Alcohol and Cigarette Use Index, a pre-registered outcome, is an additive index of the two measures, alcohol use and cigarette use, which range from 0 to 8. The week refers to the average week in the most recent month prior to Age 1 interview. The dollar expenditure on cigarettes per week is estimated by multiplying reported packs of cigarettes with the average cost of a cigarette pack by site. According to the Centers for Disease Control and Prevention, the average cost of a pack of cigarette in 2019 for the four states in BFY were: LA -$6.08, MN -$9.13, NE-$5.78, NY-$10.53. Alcohol and cigarette expenditure outcomes were winsorized at the 99^th^ percentile to adjust for extreme values. The estimated treatment effect controls for baby’s age (in months) at time of interview, whether the interview was conducted over the phone, and the following pre-registered baseline mother and child characteristics: mother’s age, completed schooling, household income, net worth, general health, mental health, race and ethnicity, marital status, number of adults in the household, number of other children born to the mother, number of cigarettes per week during pregnancy, number of drinks per week during pregnancy, father living with the mother; child’s sex, birth weight, gestational age at birth. Effect size is computed by dividing the treatment effect with the standard deviation of the full age 1 analytic samples low-cash gift group. Unadjusted p-values and pre-registered Westfall and Young adjusted p-values, which adjust for multiple hypothesis testing, are both reported. For the Westfall-Young adjustment, the substance use measures are placed into one family and the expenditure measures are placed into one family. Panel A reports on the treatment effects for the sample interviewed prior to the onset of the pandemic and Panel B reports on the full study sample. Panel B is missing substance use measures because these sensitive items were part of an audio computer-assisted survey which was administered during an in-home visit prior to the onset of the pandemic and were dropped during the pandemic when survey switched interview format from in-person to phone interviews. 1 participant with substance use measures included in panel A started the interview in person but completed the interview over the phone. Effect sizes on substance use outcomes are identical if we exclude this participant. 8 participants who were interviewed prior to the onset of the pandemic and have valid substance expenditure data were excluded from panel A because they were missing substance use measures. Including the 8 participants decreases the effect size on cigarette purchased and alcohol expenditure to 0.004 and 0.011, respectively. Standard errors are in parentheses. + p<0.10; * p<0.05; ** p<0.01.

**Table S.B4 Treatment Effect on Substance Use and Expenditure by Age 1 with ATT Weighting**

|  | **Maternal Substance Use Behavior** | | | | **Family Substance Expenditure/Purchase per Week** | | | |
| --- | --- | --- | --- | --- | --- | --- | --- | --- |
|  | Alcohol and Cigarette Use Index | Alcohol  Use | Cigarette Use | Opioid  Use | Alcohol and Cigarette (dollars) | Alcohol  (dollars) | Cigarettes (dollars) | Cigarettes  (N packs) |
| ***A. Pre-pandemic Sample*** |  |  |  |  |  |  |  |  |
| Low-cash gift group mean | 0.936 | 0.407 | 0.528 | 0.055 | 11.196 | 2.731 | 8.353 | 1.216 |
| Cash-gift treatment effect (SE) | 0.116  (0.117) | 0.091  (0.058) | 0.026  (0.090) | -0.013  (0.023) | -0.926  (2.353) | 1.267  (0.919) | -2.173  (1.975) | -0.294  (0.288) |
| Effect size | 0.082 | 0.154 | 0.021 | -0.036 | -0.035 | 0.150 | -0.094 | -0.088 |
| *p*-value, unadjusted | 0.322 | 0.121 | 0.772 | 0.567 | 0.694 | 0.169 | 0.272 | 0.309 |
| *p*-value, adjusted | 0.615 | 0.368 | 0.825 | 0.825 | 0.706 | 0.359 | 0.387 | 0.426 |
| N | 597 | 597 | 598 | 597 | 593 | 595 | 595 | 595 |
| ***B. Full Sample*** |  |  |  |  |  |  |  |  |
| Low-cash gift group mean | 0.936 | 0.407 | 0.528 | 0.055 | 9.317 | 2.947 | 6.344 | 0.897 |
| Cash-gift treatment effect (SE) | 0.116  (0.117) | 0.091  (0.058) | 0.026  (0.090) | -0.013  (0.023) | -0.287  (1.656) | 0.609  (0.713) | -0.862  (1.365) | -0.121  (0.180) |
| Effect size | 0.082 | 0.154 | 0.021 | -0.036 | -0.012 | 0.070 | -0.044 | -0.045 |
| *p*-value, unadjusted | 0.322 | 0.121 | 0.772 | 0.567 | 0.863 | 0.393 | 0.528 | 0.499 |
| *p*-value, adjusted | 0.648 | 0.419 | 0.839 | 0.839 | 0.878 | 0.681 | 0.681 | 0.681 |
| N | 597 | 597 | 598 | 597 | 920 | 922 | 927 | 927 |

Notes: Low-cash gift group observations were weighted by the odds of being in treatment to look like the treatment group observations on average. Alcohol use, cigarette use, and opioid use are each measured from a maternal report of the frequency of substance use since the baby’s birth (i.e., the last year) on a 5-point response scale (0: never in last year; 1: less than 1 time per month; 2: several times per month; 3: several times per week; 4: everyday). The Alcohol and Cigarette Use Index, a pre-registered outcome, is an additive index of the two measures, alcohol use and cigarette use, which range from 0 to 8. The week refers to the average week in the most recent month prior to Age 1 interview. The dollar expenditure on cigarettes per week is estimated by multiplying reported packs of cigarettes with the average cost of a cigarette pack by site. According to the Centers for Disease Control and Prevention, the average cost of a pack of cigarette in 2019 for the four states in BFY were: LA -$6.08, MN -$9.13, NE-$5.78, NY-$10.53. Alcohol and cigarette expenditure outcomes were winsorized at the 99^th^ percentile to adjust for extreme values. The estimated treatment effect controls for baby’s age (in months) at time of interview, whether the interview was conducted over the phone, and the following pre-registered baseline mother and child characteristics: mother’s age, completed schooling, household income, net worth, general health, mental health, race and ethnicity, marital status, number of adults in the household, number of other children born to the mother, number of cigarettes per week during pregnancy, number of drinks per week during pregnancy, father living with the mother; child’s sex, birth weight, gestational age at birth. Effect size is computed by dividing the treatment effect with the standard deviation of the full age 1 analytic samples low-cash gift group. Unadjusted p-values and pre-registered Westfall and Young adjusted p-values, which adjust for multiple hypothesis testing, are both reported. For the Westfall-Young adjustment, the substance use measures are placed into one family and the expenditure measures are placed into one family. Panel A reports on the treatment effects for the sample interviewed prior to the onset of the pandemic and Panel B reports on the full study sample. Panel B is missing substance use measures because these sensitive items were part of an audio computer-assisted survey which was administered during an in-home visit prior to the onset of the pandemic and were dropped during the pandemic when survey switched interview format from in-person to phone interviews. 1 participant with substance use measures included in panel A started the interview in person but completed the interview over the phone. Effect sizes on substance use outcomes are identical if we exclude this participant. 8 participants who were interviewed prior to the onset of the pandemic and have valid substance expenditure data were excluded from panel A because they were missing substance use measures. Including the 8 participants decreases the effect size on cigarette purchased and alcohol expenditure to 0.004 and 0.011, respectively. Standard errors are in parentheses. + p<0.10; * p<0.05; ** p<0.01.

1. Bureau of Labor Statistics. BLS [Internet]. Consumer Expenditure Survey available from <https://www.bls.gov/cex/tables/calendar-year/aggregate-group-share.htm#cu-income> [↑](#endnote-ref-2)
2. Note the number of participants who completed age-1 interviews in-person is larger than 597 (N=605 in this analytic sample) because some participants did not provide a valid substance use measure. [↑](#endnote-ref-3)
3. Ridgeway G, McCaffrey D, Morral A, Burgette L, Griffin BA. Toolkit for Weighting and Analysis of Nonequivalent Groups: A tutorial for the twang package. Santa Monica, CA: RAND Corporation. 2017 Jul 1. [↑](#endnote-ref-4)
4. Wooldridge JM. Econometric analysis of cross section and panel data. MIT press; 2010 Oct 1. [↑](#endnote-ref-5)
